# Life-Threatening Hypokalemia and Ventricular Arrhythmia Secondary to Segmental Colitis Associated With Diverticulosis: A Case Report

**DOI:** 10.7759/cureus.110852

**Published:** 2026-06-14

**Authors:** Om Jhaveri, Aaqid Syed, Cody Bulger

**Affiliations:** 1 Internal Medicine, Alabama College of Osteopathic Medicine, Dothan, USA; 2 Internal Medicine, Mobile Infirmary Medical Center, Mobile, USA

**Keywords:** chronic scad, potassium disturbances, recurrent hypokalemia, scad diagnosis, scad in men

## Abstract

Segmental colitis associated with diverticulosis (SCAD) is a localized inflammatory condition involving colonic segments affected by diverticulosis, most commonly the sigmoid colon. It is typically considered a benign entity presenting with mild hematochezia or intermittent diarrhea, with systemic complications rarely reported. We present a 47-year-old male with a history of autoimmune disease and chronic high-volume diarrhea who presented with chest pain and dyspnea. On admission, he was found to have profound hypokalemia with electrocardiographic abnormalities. His course was complicated by non-sustained ventricular tachycardia in the setting of underlying structural heart disease. An extensive infectious and inflammatory workup was unrevealing. Colonoscopy demonstrated sigmoid diverticulosis with localized peri-diverticular erythematous mucosa and rectal sparing, consistent with SCAD. Following aggressive electrolyte repletion and antibiotic therapy, the patient’s metabolic abnormalities resolved, and diarrhea improved. This case expands the clinical spectrum of SCAD by demonstrating that chronic inflammatory diarrhea may result in severe electrolyte depletion and life-threatening arrhythmias. Clinicians should consider SCAD in the differential diagnosis of chronic diarrhea, particularly when associated with unexplained electrolyte disturbances.

## Introduction

Segmental colitis associated with diverticulosis (SCAD) is a distinct clinicopathologic entity characterized by inflammation confined to colonic segments affected by diverticular disease, most commonly involving the sigmoid colon [1]. It is differentiated from diverticulitis and inflammatory bowel disease by the absence of pericolic inflammation, abscess formation, and continuous mucosal involvement with characteristic rectal sparing. Endoscopic findings typically include erythematous, friable mucosa localized to areas of diverticulosis [2]. Management is often conservative, including antibiotics, 5-aminosalicylic acid (5-ASA) agents, or both. Systemic complications are uncommon and rarely described in the literature.

SCAD generally follows a benign clinical course with patients presenting with mild symptoms such as abdominal discomfort, diarrhea, or hematochezia. Although diarrhea is a common presenting feature of SCAD, it is typically mild. However, when persistent or high-volume, chronic diarrhea can result in substantial electrolyte losses leading to potentially life-threatening conditions [3]. We present an unusual case of SCAD that presented with profound hypokalemia secondary to chronic diarrhea, leading to potentially fatal ventricular arrhythmias [4]. We present a case of a 47-year-old, African American male with the past medical history of ankylosing spondylitis, rheumatoid arthritis, psoriasis, chronic alcohol use disorder, chronic macrocytic anemia, and chronic high-volume diarrhea, who presented with chest pain, dyspnea, and neuromuscular symptoms. This case highlights the diagnostic difficulty in recognizing SCAD as the underlying cause of chronic diarrhea.

## Case presentation

A 47-year-old male presented to the emergency department with one month of progressive chest pain, dyspnea, generalized weakness, and bilateral lower extremity edema. His symptoms were associated with perioral numbness and distal paresthesia. The patient had recently been started on oral potassium supplementation for hypokalemia identified by his PCP. However, his symptoms continue to worsen despite adherence. The patient also reported a greater than 20-year history of chronic watery diarrhea, averaging 10-20 non-bloody bowel movements daily. There was no history of hematochezia, melena, abdominal pain, fevers, recent travel, or antibiotic use.

His medical history was significant for ankylosing spondylitis with multisystem involvement, rheumatoid arthritis, psoriasis, chronic macrocytic anemia, hypertension, and mitral regurgitation. He also had a history of chronic alcohol use (approximately 21 drinks per week) and remote tobacco use. Prior immunosuppressive therapy included adalimumab, which had been discontinued due to hematologic abnormalities, and methotrexate, which was stopped due to intolerance.

He had a prior gastric ulcer perforation in 2017.

Approximately one year prior to presentation, the patient was worked up for marked thrombocytosis, along with leukocytosis and macrocytosis. Bone marrow evaluation demonstrated trilineage hematopoiesis without evidence of a myeloproliferative neoplasm. Cytogenetic and molecular studies were unrevealing, and findings were ultimately attributed to reactive thrombocytosis.

On presentation, laboratory evaluation revealed severe electrolyte derangements, including profound hypokalemia, hypomagnesemia, hypocalcemia, lactic acidosis, and acute kidney injury as summarized in Table 1. Troponin and NT-proBNP were elevated. Electrocardiography demonstrated sinus rhythm with QT prolongation and ventricular ectopy. CT angiography of the chest showed mild pulmonary edema without evidence of pulmonary embolism. He was admitted to the intensive care unit for management of severe hypokalemia.

**Table 1:** Key summary of laboratory evaluation of patient on arrival to the emergency department.

Laboratory Test	Patient Value	Normal Reference Range	Interpretation
Potassium (mmol/L)	1.7	3.5-5.0	Critically low
Magnesium (mg/dL)	1.6	1.7-2.2	Low
Calcium, total (mg/dL)	6	8.6-10.2	Severely low
Lactic acid (mmol/L)	6.6	0.5-2.0	Markedly elevated
Creatinine (mg/dL)	1.84	0.7-1.3	Elevated (acute kidney injury (AKI))
White blood cell count (×10⁹/L)	13.9	4.0-11.0	Elevated
Hemoglobin (g/dL)	10.7	13.5-17.5	Low (anemia)
Mean corpuscular volume (fL)	116	80-100	Elevated (macrocytosis)
NT-proBNP (pg/mL)	705	<125 (<50 years)	Elevated
Troponin (ng/L)	160 (peak)	<14 (lab dependent)	Elevated
Serum ethanol (mg/dL)	130	0	Elevated

Electrocardiography (Figure 1) demonstrated a sinus rhythm with frequent atrial premature complexes, including couplets, and intermittent sinus pauses. There was evidence of left ventricular hypertrophy with secondary repolarization abnormalities. Lateral Q waves and mild ST-segment depression in the anterolateral leads raised concern for possible ischemia. The QT interval was prolonged, with QTc measurements exceeding 475-500 ms.

**Figure 1:**
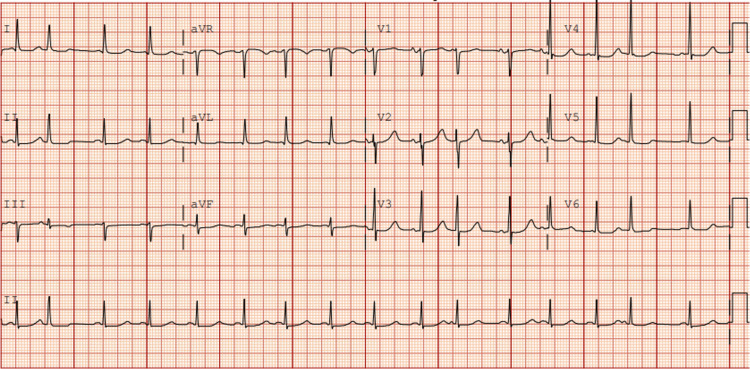
Electrocardiogram from ICU demonstrating sinus rhythm with atrial premature complexes, prolonged QTc (>500 ms), sinus pauses, and anterolateral ST-segment depression associated with profound electrolyte derangements.

In the ICU, the patient received aggressive intravenous electrolyte repletion, including potassium, magnesium, and calcium, along with intravenous fluids. His metabolic derangements and renal function improved with treatment. On hospital day 3, telemetry revealed an eight-beat run of non-sustained ventricular tachycardia. No sustained arrhythmias occurred following correction of electrolyte disturbances. A transthoracic echocardiography (TTE) was performed, and it demonstrated a preserved left ventricular ejection fraction of 55 to 60% with mild to moderate tricuspid regurgitation and moderate mitral regurgitation. After this, transesophageal echocardiography confirmed moderate-to-severe mitral regurgitation. Given that the patient was hemodynamically stable and had an absence of refractory symptoms, surgical intervention was deferred in favor of optimized medical therapy, and a repeat TTE was planned in three months.

Given his long-standing diarrhea and severe electrolyte abnormalities, an extensive gastrointestinal evaluation was performed. Stool studies, including *Clostridioides difficile* testing, gastrointestinal pathogen PCR panel, stool cultures, Shiga toxin assay, fecal calprotectin, and fecal leukocytes, were negative. Computed tomography of the abdomen and pelvis demonstrated diverticulosis without evidence of obstruction or inflammatory mass (Figures 2-3). Colonoscopy without ileal intubation revealed diverticulosis involving the sigmoid and descending colon with localized erythematous mucosa surrounding diverticular orifices, consistent with SCAD (Figure 4). The rectum and proximal colon were normal. Biopsies demonstrated localized inflammatory changes without features of diffuse inflammatory bowel disease. Histopathologic examination of the ascending colon biopsy demonstrated focal active inflammation with mildly increased chronic inflammatory infiltrates within the lamina propria. No crypt architectural distortion, granulomas, dysplasia, or well-established features of chronic inflammatory bowel disease were identified. Descending colon biopsies demonstrated reactive colonic mucosa without significant histopathologic abnormality.

**Figure 2:**
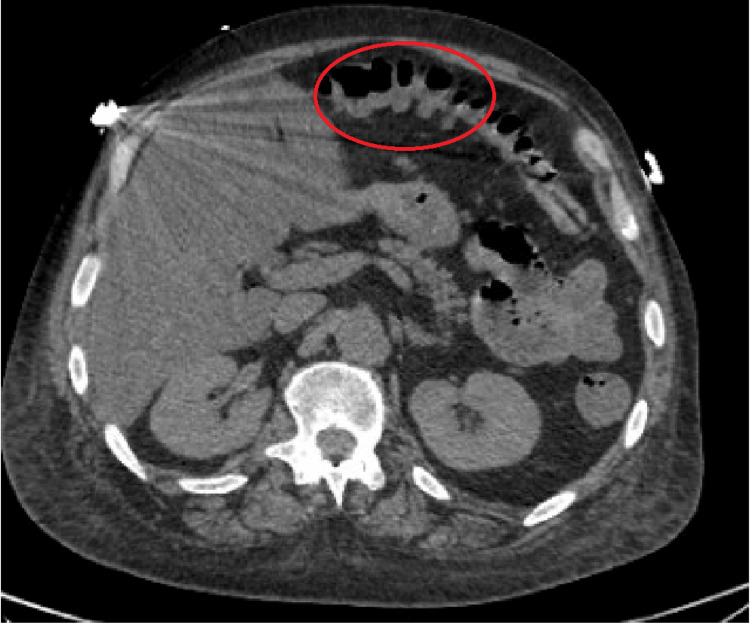
Axial computed tomography image of the abdomen demonstrating scattered colonic diverticulosis (red oval) without evidence of acute diverticulitis.

**Figure 3:**
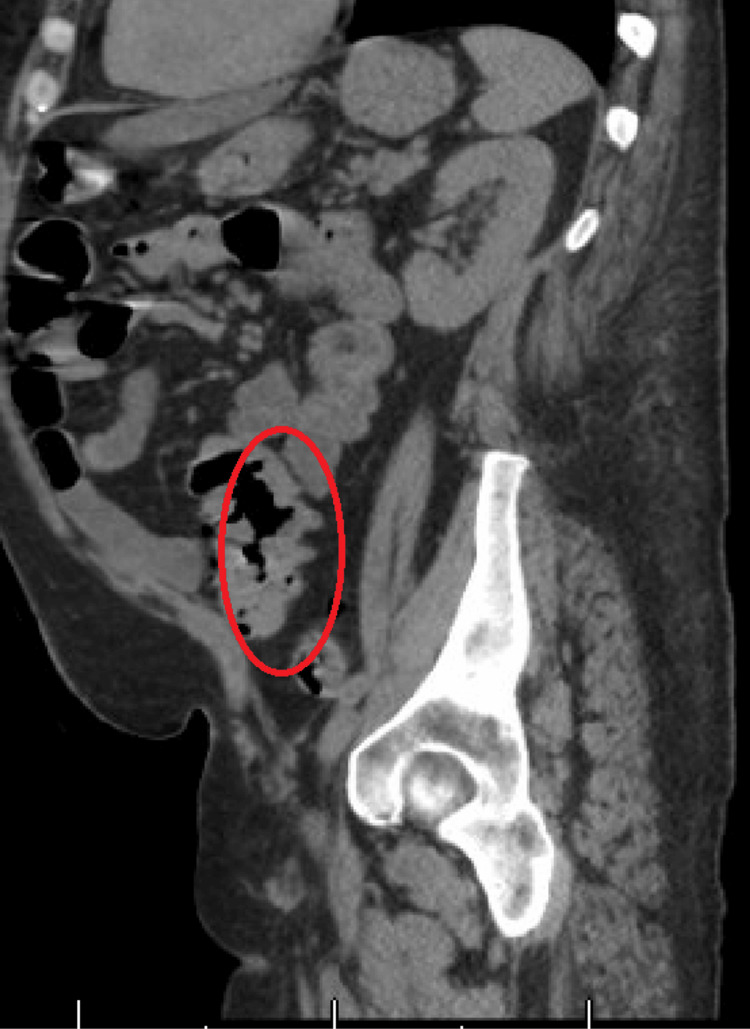
Sagittal computed tomography image demonstrating diverticular outpouchings within the sigmoid colon (red oval), corresponding to the region of segmental colitis identified on colonoscopy.

**Figure 4:**
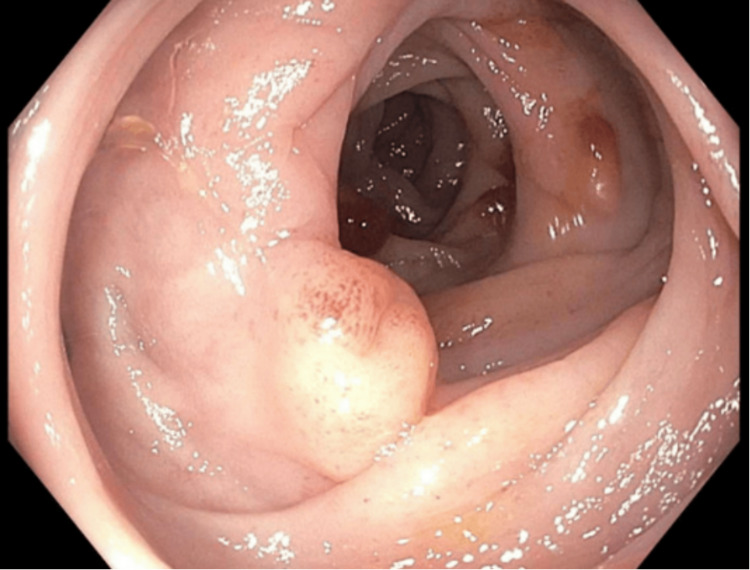
Colonoscopic image demonstrating sigmoid diverticulosis with localized peri-diverticular erythema and erythematous mucosal changes consistent with segmental colitis associated with diverticulosis (SCAD).

Sigmoid colon showed scattered medium-mouthed diverticulosis with localized peri-diverticular erythema and moderately erythematous mucosa (Figures 4-5). Localized peri-diverticular inflammatory changes and erythematous mucosa consistent with SCAD were observed within the sigmoid colon (Figures 4-5). The transverse colon demonstrated preserved vascular architecture without inflammatory change (Figure 6).

**Figure 5:**
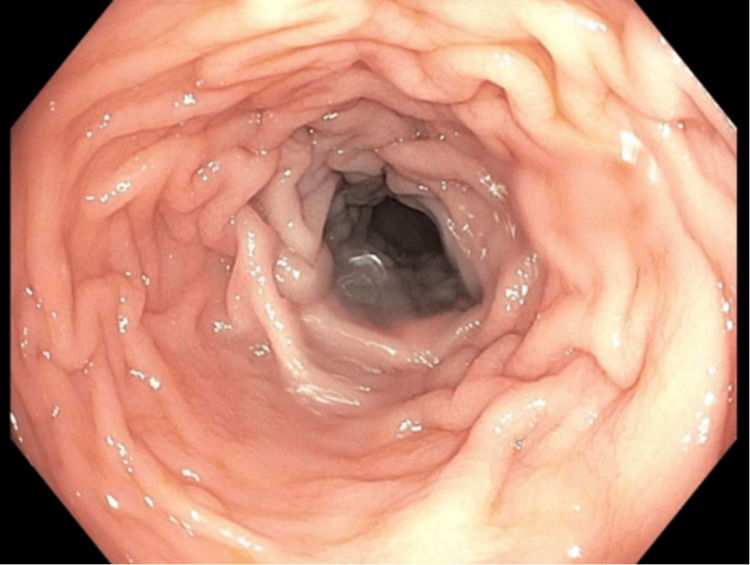
Colonoscopic image of the sigmoid colon demonstrating diffuse mucosal erythema and inflammatory changes surrounding diverticular openings, consistent with segmental colitis associated with diverticulosis (SCAD).

**Figure 6:**
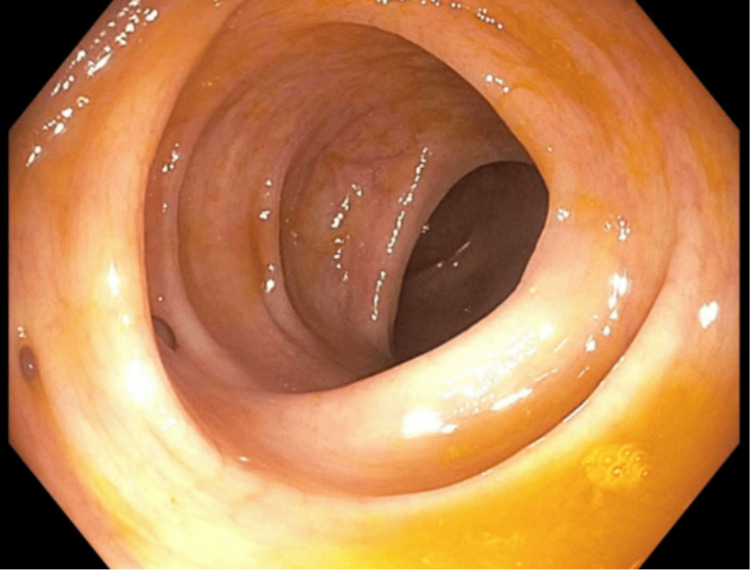
Colonoscopic image of the transverse colon demonstrating preserved vascular pattern and normal-appearing mucosa, supporting proximal colonic sparing.

The patient was treated with a 10-day course of ciprofloxacin and metronidazole. Mesalamine therapy was planned if symptoms persisted. Following treatment initiation, his diarrhea improved significantly to three to four bowel movements daily. Electrolytes were normalized prior to discharge, and acute kidney injury had resolved. His macrocytic anemia persisted without evidence of nutritional deficiency and was attributed to chronic alcohol use and prior methotrexate exposure. He was discharged with multidisciplinary outpatient follow-up. At approximately eight weeks following discharge, laboratory studies demonstrated sustained correction of electrolyte abnormalities with potassium of 3.9 mmol/L, magnesium of 2.1 mg/dL, creatinine of 1.44 mg/dL, and hemoglobin of 11.7 g/dL. The patient reported intermittent diarrhea but had not yet completed gastroenterology follow-up.

## Discussion

This case represents an uncommon systemic manifestation of SCAD, characterized by severe electrolyte derangements and ventricular arrhythmia requiring intensive care management. Although SCAD is generally considered a localized inflammatory condition with a benign clinical course, this presentation broadens its clinical spectrum and underscores the potential consequences of prolonged, unrecognized inflammatory diarrhea.

The patient's longstanding high-volume diarrhea likely resulted in chronic gastrointestinal potassium wasting and progressive total body potassium depletion. Colonic inflammation has been shown to enhance active potassium secretion [5]. The severity of hypokalemia in this case suggests significant total body depletion rather than redistribution alone. Concomitant hypomagnesemia further impaired renal potassium reabsorption, limiting effective correction and increasing susceptibility to cardiac arrhythmias [6].

Cardiac susceptibility played a critical role in this presentation. Structural heart disease, including moderate-to-severe mitral regurgitation, likely lowered the threshold for arrhythmogenesis. Severe hypokalemia predisposes to QT prolongation and ventricular arrhythmias, while hypomagnesemia further destabilizes myocardial ion channel function [7,8].

The diagnostic process was further complicated by the patient's underlying autoimmune conditions. Ankylosing spondylitis, rheumatoid arthritis, and psoriasis are associated with an increased prevalence of inflammatory bowel disease, often leading to diagnostic anchoring toward Crohn's disease or ulcerative colitis in patients with chronic diarrhea [9,10]. However, the presence of rectal sparing and inflammation localized to diverticular segments on colonoscopy, along with negative stool studies and fecal calprotectin, supported a diagnosis of SCAD rather than diffuse inflammatory bowel disease. This highlights the importance of avoiding diagnostic anchoring, especially in patients with multiple autoimmune diagnoses.

Long-term biologic therapy may also predispose patients to infectious gastrointestinal complications, although extensive infectious evaluation, including stool culture, *Clostridioides difficile* testing, GI BioFire panel, Shiga toxin testing, and fecal leukocyte assessment, was unrevealing. Chronic alcohol use represented another potential contributor through mechanisms including intestinal mucosal injury, altered gut motility, nutritional deficiencies, and possible exocrine pancreatic dysfunction. Although dedicated pancreatic function testing was not performed, CT imaging demonstrated a normal-appearing pancreas, and serum lipase levels remained within normal limits. Medication-induced diarrhea, microscopic colitis, and other malabsorptive disorders were also considered. Ultimately, the characteristic endoscopic distribution of inflammation localized to diverticular-bearing segments of the sigmoid colon, combined with negative infectious studies and nonspecific histopathology, favored SCAD as the most likely diagnosis.

The previous hematology evaluation is not worthy in this patient's case. The extreme thrombocytosis prompted a thorough investigation for myeloproliferative neoplasm. It included bone marrow aspirate, cytogenetic analysis, FISH panels, and flow cytometry. After the discontinuation of anagrelide, there was an absence of clonal markers and a normalization of platelet counts, which supported reactive thrombocytosis. Chronic inflammatory conditions are well recognized as drivers of secondary thrombocytosis. Therefore, it is plausible that the chronic colonic inflammation contributed to the systemic inflammatory signaling and the elevation in platelet production. While definitive causality cannot be established, the temporal association and absence of clonal markers favor this reactive process.

The patient's metabolic derangements were likely multifactorial. Chronic alcohol use may have contributed to magnesium depletion, macrocytosis, and increased arrhythmogenic risk, while also potentially exacerbating diarrheal losses [6]. Acute kidney injury likely resulted from volume depletion and transient renal hypoperfusion, further impairing electrolyte homeostasis [11].

The convergence of multiple chronic conditions culminating in an acute life-threatening event highlights the uniqueness of this case. From a clinical perspective, this case emphasizes the importance of evaluating chronic diarrhea even when it is longstanding and previously tolerated. Persistent electrolyte losses may remain subclinical until decompensation occurs. In patients with underlying cardiac disease, even moderate electrolyte disturbances may precipitate significant arrhythmias. Clinicians should avoid attributing chronic diarrhea in autoimmune patients solely to inflammatory bowel disease without endoscopic confirmation, as alternative entities such as SCAD may be responsible.

## Conclusions

SCAD is typically a benign, localized inflammatory condition. However, this case demonstrates that it can rarely present with severe systemic complications, including profound hypokalemia and life-threatening arrhythmias. Chronic inflammatory diarrhea may result in significant electrolyte depletion, particularly in patients with underlying cardiac disease or concomitant metabolic risk factors. Recognition of SCAD as a potential etiology of chronic diarrhea is essential to avoid diagnostic delay and prevent serious complications.
